# The Bacterial Control of Poly (Lactic Acid) Nanofibers Loaded with Plant-Derived Monoterpenoids via Emulsion Electrospinning

**DOI:** 10.3390/polym13193405

**Published:** 2021-10-03

**Authors:** Tahmineh Hemmatian, Kwon Ho Seo, Meltem Yanilmaz, Juran Kim

**Affiliations:** 1 Advanced Textile R&D Department, Korea Institute of Industrial Technology (KITECH), Ansan 15588, Korea; hanh11@snu.ac.kr (T.H.); tjrnjsgh@kitech.re.kr (K.H.S.); 2 Textile Engineering, Istanbul Technical University, Istanbul 34467, Turkey; yanilmaz@itu.edu.tr

**Keywords:** monoterpenoid, carvacrol, geraniol, *Escherichia coli*, *Staphylococcus epidermidis*, poly (lactic acid), antibacterial, personal protective textiles

## Abstract

Plant-derived monoterpenoids have been shown to possess various biological effects, providing a scientific basis for their potential usage as antibacterial agents. Therefore, considering problems surrounding bacteria′s antibacterial resistance, the utilization of natural antimicrobial compounds such as monoterpenoids in different industries has gained much attention. The aim of this study was to fabricate and characterize various concentrations of plant-derived monoterpenoids, geraniol (G) and carvacrol (C), loaded into poly(lactic acid) (PLA) nanofibers via emulsion electrospinning. The antibacterial activities of the fabricated nanofibers were evaluated using three types of antibacterial assays (inhibition zone tests, live/dead bacterial cell assays, and antibacterial kinetic growth assays). Among the samples, 10 wt% carvacrol-loaded PLA nanofibers (C10) had the most bactericidal activity, with the widest inhibition zone of 5.26 cm and the highest visible dead bacteria using the inhibition zone test and live/dead bacterial cell assay. In order to quantitatively analyze the antibacterial activities of 5 wt% carvacrol-loaded PLA nanofibers (C5), C10, 5 wt% geraniol-loaded PLA nanofibers (G5), and 10 wt% geraniol-loaded PLA nanofibers (G10) against *E. coli* and *S.* *epidermidis*, growth kinetic curves were analyzed using OD_600_. For the results, we found that the antibacterial performance was as follows: C10 > C5 > G10 > G5. Overall, carvacrol or geraniol-loaded PLA nanofibers are promising antibacterial materials for improving fiber functionality.

## 1. Introduction

Antimicrobial agents play a critical role in reducing infectious diseases by acting against bacterial infections. However, as a result of decades of using antimicrobial agents such as antibiotics, coping with the antibiotic resistance of bacteria has become a great concern for global public health investigations of new antimicrobial compounds [1], as well as the global health community [2,3]. Gram-positive *Staphylococcus epidermidis* (*S. epidermidis*) and Gram-negative *Escherichia coli* (*E. coli*) are well-known bacteria that establish chronic infections. One of the most common bacterial species that is widely present on human skin is *S. epidermidis* [4,5]: the leading source of about two-thirds of human infections and a serious threat to human life [4,6,7] as it belongs to the group of coagulase-negative staphylococci (CNS) [8]. CNS causes many infections such as bacteremia, central nervous system shunt infection, endocarditis, urinary tract infection, surgical site infections, endophthalmitis, and foreign body infection [5]. On the other hand, *E. coli*, the Gram-negative strain, is commonly found in the large intestine of humans and other warm-blooded animals [9]. Most *E. coli* strains in the human intestine are harmless, while pathogenic *E. coli* strains outside of their natural habitat can cause various types of extraintestinal infections [10] such as enteritis, urinary tract infection, septicaemia, neonatal meningitis, and many other clinical infections [11].

To prevent and/or restrict the growth of bacteria, yeasts, and mold, there have been notable investigations revolving around monoterpenoids and the potential ability of plant extracts to act as alternative agents modifying bacterial resistance [1,12,13]. Several reports have stated that the hydrophobic terpenoids or phenolic compounds content of monoterpenoids can penetrate the cell membrane, resulting in disruption, synthesis, or cell lysis [14,15,16,17,18]. Monoterpenoids consist of 10 carbon backbones or 2 isoprene unit structures, such as carvacrol and geraniol. Carvacrol, which is found in many aromatic plants from the *Lamiaceae* family, is known for its wide spectrum of biological effects [19]. Many studies to this day have reported on carvacrol′s notable inhibition against *E. coli*, *Staphylococcus aureus* (*S. aureus*), and *S. epidermidis* [20,21]. Geraniol, which is found in monoterpenoids of rose and lemongrass [22], is used for its flavor and odor [23] and is also well known for its antifungal and antibacterial activities such as its bactericidal effects against *S. aureus*, *S. epidermidis*, and *E. coli* [23,24,25,26,27,28]. In a previous disk method study by Kim et al. [28], carvacrol and geraniol showed a dose-related increase in zone of inhibition against five tester strains including *E. coli* as a 10% dose of carvacrol, and geraniol showed a higher inhabitation potential against the bacterial strains compared to a 5% dose. According to Ultee et al. [29], the hydroxyl group and the presence of a delocalized electrons system in phenolic compounds such as carvacrol are reported to act as a proton exchanger by reducing the gradient across the cytoplasmic membrane. The resulting collapse of the proton motive force and depletion of the ATP pool eventually lead to cell death. Carvacrol and geraniol are both plant-derived monoterpenoids, and the main difference between them is the presence of a phenolic ring in the carvacrol chemical structure. Therefore, the importance of a delocalized electron system allowing the hydroxyl group to release its proton can be observed through investigating the antibacterial effect of carvacrol and geraniol.

Naturally derived polymers such as poly (lactic acid) (PLA) have been subjects of interest in stark contrast to synthetic polymers and their environmental problems. Due to their biodegradable nature, high safety, mechanical strength, fabricability, and biocompatibility, PLA has presented new biomedical and food packaging possibilities [30,31,32]. PLA is also the first substance that has successfully made the link between natural and synthetic fibers [33] and has been reported to be an efficient stabilizer-carrier for various kinds of molecules such as monoterpenoids, thereby offering innovative solutions for wound healing or as a coating for medical devices [34]. For the development of further innovative solutions such as advanced dressings capable of promoting rapid and efficient wound healing, there have been proposals to utilize emulsion electrospinning techniques as a new green approach for utilizing liquids such as monoterpenoids in nanofibers through incorporating monoterpenoids with bioactive agents found in nanofibers in terms of environments [35,36,37,38,39]. These fibers that have been developed via encapsulating systems, along with micro- and nanoparticles, capsules, droplets, and cyclodextrin complexes, have been used to control the properties of the resulting fibers and achieve bioactivity of monoterpenoids. Nanofibers obtained through electrospinning techniques have a high surface-to-volume ratio, small fiber diameter, and high microscale porosity [40]. The high surface-to-volume ratio and microscale porosity result in the controlled release of monoterpenoids, which has been proven to reduce the cytotoxic effects of some monoterpenoid components in human cells [41]. Although volatile active compounds such as plant-derived monoterpenoids easily degrade into other substances due to high temperatures, light, oxygen, and moisture [41], electrospinning techniques do not require high temperatures; as a result, characteristics of the monoterpenoids are maintained even after they are mixed in different polymeric systems, and they can still possess antimicrobial activity after their release from the electrospun fibers.

Textiles have recently been used in fibrous materials for a wide range of applications in medicine and hygiene, including health care and hygiene coatings, medical devices, water purification systems, and air filters [42,43,44,45]. Consequently, the use of antimicrobial textiles for functionality has become an attractive idea because microorganism activity in textiles is detrimental to both the user and textile itself. For example, *E. coli* and *S. aureus*, along with many other bacteria involved in textile contamination, can cause pathogenic effects. Thus, interests in utilizing antimicrobial fabrics have been increasing [46]. Disposal personal care products as protective barriers have been used for many decades to protect personal health when exposed to pathogens. Personal care products have become an everyday necessity to protect from biohazards, but the disposal of personal care products has become a considerable global waste problem caused by an exponential increase in the usage of disposable fibrous personal care products. In order to solve this problem, we investigated biodegradable PLA nanofibers-loaded plant-derived monoterpenoids (carvacrol and geraniol) and evaluated the antibacterial effects against *E. coli* and *S. epidermidis.* This study suggested that monoterpenoid-loaded PLA nanofibers can serve as a replacement for commercial synthetic disposal fibrous products in eliminating biohazardous bacteria, leading to positive impacts on the human health and environment.

The inclusion of novel, antibacterial leading compounds such as monoterpenoids in biodegradable polymeric fibers by integrating antibacterial properties in hygiene textile products such as masks, wipes, hospital gowns, and surgical covers was a notable method to evolve this interest. Therefore, the aim of this study was to investigate the antibacterial effects of several compounds present in monoterpenoids utilized in biodegradable PLA electrospun nanofibers and to study the bactericidal effects of carvacrol and geraniol in biodegradable PLA nanofibers against *E. coli* and *S. epidermidis*.

## 2. Materials and Methods

Poly(lactic acid) granules were obtained from Goodfellow (Goodfellow Cambridge Limited, Huntingdon, UK). Gram-negative strain *Escherichia coli* KCTC 1039 (*E. coli*) and Gram-positive strain *Staphylococcus epidermidis* KCTC 13172 (*S. epidermidis*) used as model bacteria were purchased from KCTC (Korean Collection for Type Cultures, Jeongyep-si, Korea). Geraniol, carvacrol, dimethylformamide (DMF), sodium chloride (NaCl), mannitol salt phenol red agar, tryptic soy broth, osmium tetroxide (4% *w/v*), and dichloromethane (DCM) were purchased from Sigma-Aldrich (St. Louis, MO, USA). Tryptic soy agar and MacConkey sorbitol agar were purchased from Becton Dickinson (BD Difco^TM^, Sparks, MD, USA). Phosphate-buffered saline (PBS, pH 7.4) and LIVE/DEAD^®^ BacLight^TM^ Bacterial Viability Kit L-7007 (molecular probes) were supplied by Thermo Fisher Scientific (Thermo Fisher Scientific Inc., Waltham, MA, USA).

### 2.1. Preparation of Electrospinning Webs

**Figure 1:**
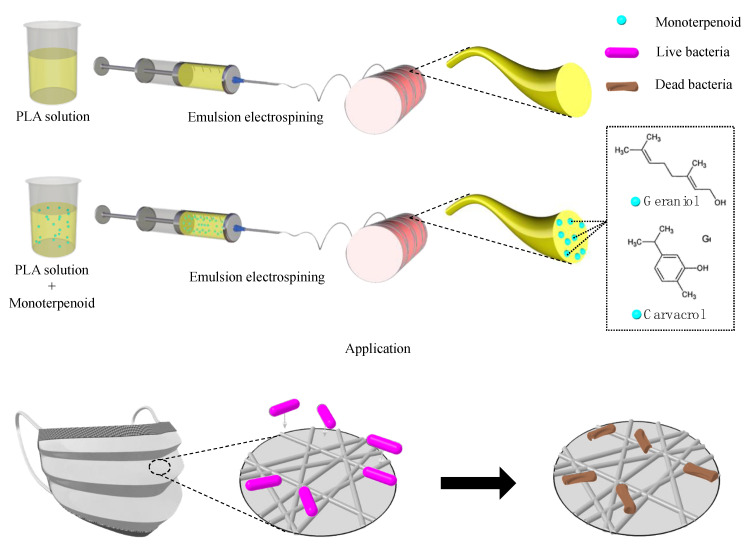
Schematic illustration of the emulsion electrospinning procedure.

The electrospun webs were prepared by emulsion electrospinning. A 10% (*w*/*v*) PLA solution with a 1:1 volume ratio of DCM and DMF was prepared, and the control PLA webs were electrospun at a feeding rate of 3 mL h^−1^ at 19 kV with the needle gauge of 18 G. The tip-to-collector distance was 15 cm. Monoterpenoid-loaded PLA webs were prepared by adding various concentrations of monoterpenoids into the prepared 10% (*w*/*v*) PLA solution (5 and 10% based on the weight of solution). For homogenization, the PLA–oil solution was stirred with a magnetic stirrer bar at room temperature for 5 min followed by 10 min of sonication (Power sonic 510, Seoul, Korea). Then, the solution was electrospun at a feeding rate of 2 mL h^−1^ and voltage of 19 kV. An 18-gauge needle was used with the tip-to-collector distance remaining at 15 cm. The preparation of the electrospun webs is illustrated in Figure 1.

### 2.2. Characterization and Measurements

#### 2.2.1. Surface Morphology and Fiber Diameter 

The morphology and fiber diameter of the electrospun webs were analyzed by scanning via an electron microscope (Hitachi, Tokyo, Japan). The mean fiber diameter and distribution were estimated by measuring at least fifty fibers from SEM images using Nahwoo imaging software (iworks 2.0, Suwon, Korea). Sample coding is presented in Table 1.

#### 2.2.2. Thermogravimetric Analysis (TGA)

TGA analysis was performed to evaluate the thermal stability of electrospun webs using a regular Thermogravimetric Analyzer instrument (TGA Q500) from TA Instruments. Measurements were carried out under a nitrogen atmosphere with a heating rate of 20 °C/min from 25 to 500 °C.

#### 2.2.3. Fourier Transform-Infrared Spectroscopy (FT-IR)

FT-IR spectroscopy (Nexus670, Gaithersburg, MD, USA) was used to analyze the composition of the materials and the produced nanofiber webs. FTIR spectra were recorded in the 500–4000 cm^−1^ range by means of KBr pellets at a controlled ambient temperature of 25 °C.

#### 2.2.4. Viscosity and Conductivity of PLA Solutions

The viscosities were measured at 25 °C using a Brookfield RVT rheometer (Toronto, ON, Canada). The electrical conductivities were measured at 25 °C using a conductometer InoLab (Weilheim, Germany).

### 2.3. Antibacterial Assay

The qualitative antibacterial activity of electrospun webs were evaluated according to standard AATCC 147-1998 through a disc diffusion assay against *E. coli* and *S. epidermidis*. First, 1 mL of overnight-precultured bacterial inoculums in nutrient broth was transferred to a new 10 mL fresh broth and incubated for 5 h at 200 rpm at 37 °C. The cultures were then diluted to an OD_600_ of 0.7 corresponding to 3.5 × 10^9^ and 1.8 × 10^9^ CFU mL^−1^ for *E. coli* and *S. epidermidis*, respectively, via a microplate reader (Allsheng, Hangzhou, China). The diluted culture was then spread over the entire surface of the nutrient agar plate using a spreader. Electrospun webs were cut into 20 mm diameter discs and then placed on the inoculated nutritional agar plate’s surface. The antimicrobial activity of the electrospun webs were recorded in terms of inhibition zone diameter (mm) using a manual caliper after plates were incubated for 24 h at 37 °C. Experiments were replicated for each sample. For the morphology of bacteria, samples were observed by SEM. Bacteria cells were fixed by osmium tetroxide vapor (2% *w*/*v*) for 24 h.

### 2.4. Cell Viability Aassay

The LIVE/DEAD^®^ BacLight^TM^ Bacterial Viability Kit assay was performed to visualize the presence of live and dead bacteria cells on the electrospun webs. For the purpose of this study, 5 mm × 5 mm cut electrospun webs were fixed on 5 mm × 5 mm glasses. Then, oxygen plasma treatment was performed at 200 W and 160 sccm for 2 min to improve the webs’ wettability using a plasma system (Femto Science, Hwaseong, Korea). An amount of 50 μL of previously made fresh *E. coli* bacteria culture was transferred to a well plate containing the samples. After an hour of incubation at 37 °C, 950 μL of fresh nutrient broth was added to the wells and the plates were incubated for another 23 h. Samples were then rinsed 2× in 250 μL of 0.85% NaCl solution. To stain using the Live/Dead assay, 10 μL of each reagent mix A (fluorescent nucleic acid stains SYTO) and reagent mix B (propidium iodide (PI)) from the kit were added to the samples in the 96-well plate and incubated at room temperature for 15 min, following the instructions of the manufacturer. Samples were imaged with a confocal laser scanning microscope using a 40x water lens (LSM700, Zeiss, Oberkochen, Germany).

### 2.5. Quantitative Antibacterial Growth Kinetic Curves Assay

Antibacterial activity of PLA nanofibers loaded with carvacrol and geraniol were evaluated using kinetic growth curves by measuring OD_600_. Each electrospun PLA web was prepared (20 × 20 mm) and added in 10 mL of bacterial suspension at 7.8 × 10^6^ or 2.12 × 10^7^ CFU mL^−1^ for *E. coli* or *S. epidermidis*, respectively. Then, they were cultured for 72 h in a shaking incubator at 150 rpm at 37 °C. The growth kinetic curves were plotted by measuring OD_600_ for a 72 h incubation time every 2 h.

## 3. Results and Discussion

### 3.1. Morphology of PLA Nanofibers

**Figure 2:**
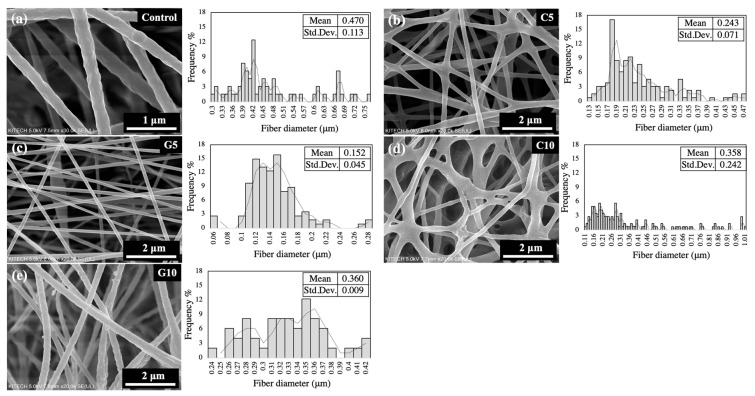
The morphology and nanofiber diameter distributions of electrospun webs with different concentrations of monoterpenoids: (**a**) control, (**b**) C5, (**c**) G5, (**d**) C10, and (**e**) G10.

Pure PLA (control) and oil-loaded PLA webs (C5, C10, G5, and G10) were fabricated by the electrospinning method. Prepared webs were coded as shown in Table 1. Figure 2 represents the morphology of the electrospun webs, as well as their mean fiber diameter and distribution. The electrospun pure PLA web exhibited a smooth surface and uniform diameters along their lengths, while the addition of monoterpenoids showed a marginal effect on fiber diameter and shape. The average diameter of the control web was 0.470 ± 0.113 µm. The addition of monoterpenoids (5 wt%) resulted in fibers with thinner diameters (0.152 ± 0.045 and 0.243, 0.071 µm in G5 and C5, respectively). With the monoterpenoids content increased up to 10 wt%, the fiber diameter increased to 0.360 ± 0.009 and 0.000 ± 0.009 µm in G10 and C10, respectively. Therefore, the greater number of cross-linking in fibers loaded with 10 wt% of the component compared to 5 wt% shows fiber dependence on the amount of monoterpenoids. According to the SEM images, adding carvacrol to the PLA solution may result in physical cross-linking among fibers. Previous studies have also reported that cross-links in the fiber may be changed by some components of monoterpenoids [47,48]. The study about the variations in fiber morphology reported that the solution viscosity and the electrical conductivity were critical parameters to influence the fiber morphology such as beads formation of the fibers. However, in this study, there was little relationship between the fiber dimeters and addition of monoterpenoids. The results showed a similar viscosity of 36–40 mPa·s and conductivity of 0.035~0.04 μS cm^−1^. A further detailed study of the relationship between fiber morphology and viscosity (or conductivity) of the PLA solution is required.

### 3.2. Thermogravimetric Analysis (TGA)

**Figure 3:**
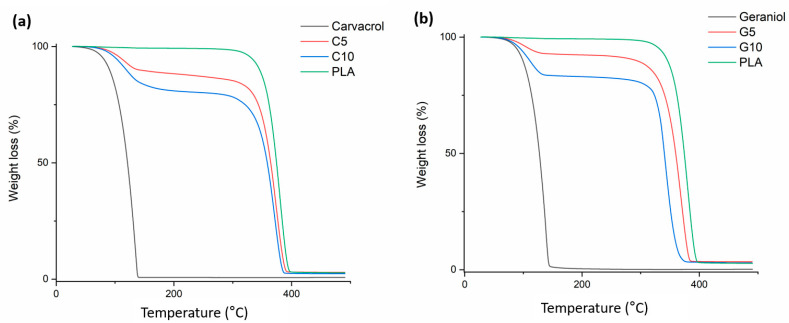
TGA diagrams for (**a**) neat PLA nanofibers (control), C5, C10, and carvacrol (C); and (**b**) neat PLA nanofibers (control), G5, G10, and geraniol (G).

The TGA results provided quantitative information on the thermal stability of monoterpenoids in monoterpenoid-loaded webs, as summarized in Table 2. To compare the TGA thermograms of the control electrospun web, G and C are also presented (Figure 3). As observed in the TGA thermogram (Figure 3), the one-step weight loss of 99.26% and 99.77%, from 27 to 133 °C and 22 to 133 °C, corresponded to the evaporation of pure C and G, respectively (Table 3). As shown in Figure 3a, the TGA curve for the control electrospun web showed a one-step degradation with a major weight loss of 96% from 257 to 375 °C, corresponding to the degradation of PLA through the loss of ester groups [49,50]. In contrast, the addition of monoterpenoids to PLA fibers displayed two degradation steps, associated with the breakdown of the PLA polymer: 1. Between 27 and 150 °C with a weight loss of around 7–19%, representing the evaporation of loaded monoterpenoids; 2. Between 150 and 374 °C and a weight loss ranging from 77 to 90%. A significant increase in the rate of weight loss at the first stage was observed when the content of monoterpenoids such as carvacrol and geraniol was increased from 5 wt% to 10 wt%. Therefore, it can be understood that the increased amount of monoterpenoids was successfully loaded into the nanofiber.

### 3.3. FT-IR Spectroscopy

**Figure 4:**
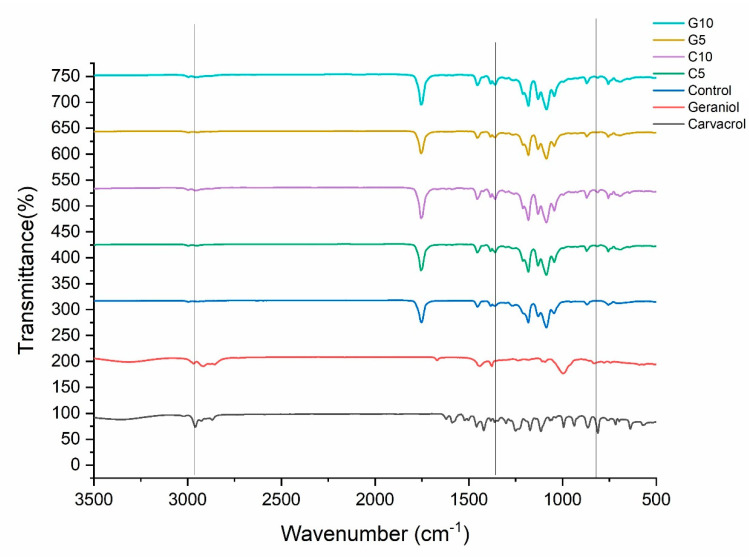
FT-IR profiles of neat PLA nanofibers (control), C5, C10, carvacrol, G5, G10, and geraniol.

The infrared spectra of the carvacrol, geraniol, pure electrospun PLA fiber, carvacrol-loaded electrospun PLA fibers, and geraniol-loaded electrospun PLA fibers are shown in Figure 4. The spectra of carvacrol and geraniol showed the stretching absorption peak at 3355 cm^−1^ and 3319 cm^−1^, respectively, associated with the –OH group. The peaks at 2958 cm^−1^ and 2914 cm^−1^ could be related to C–H stretching bonds. The bands between 1620 cm^−1^ and 1420 cm^−1^ in carvacrol and 1668 cm^−1^ and 1440 cm^−1^ in geraniol could be attributed to C–C stretching of the aromatic ring of monoterpenoids. The C=C stretching of the aromatic ring was found at 864 cm^−1^ and 811 cm^−1^ for carvacrol and 1666 cm^−1^ for geraniol. The PLA spectrum showed a peak at 1751 cm^−1^ related to the C=O stretching of carbonyl groups of PLA. The peaks at 1182 cm^−1^ and 1085 cm^−1^ were assigned to C–O–C bending vibrations, while the peak at 1044 cm^−1^ was due to C–CH_3_ vibrations. The observed peaks at 1453 cm^−1^ and 1382 cm^−1^ were attributed to the C–H deformation from –CH_2_. The C–C stretching vibrations were identified at 870 cm^−1^ and 756 cm^−1^. PLA’s amorphous and crystalline structure was associated with absorption bands at 862 cm^−1^ and 755 cm^−1^. The peak position at 3355 cm^−1^ shifted to around 3500 cm^−1^ in PLA fibers as the carvacrol and geraniol content increased from 0 to 10%. The peak of carvacrol at 1620 cm^−1^ appeared in carvacrol-loaded PLA fiber at 1620 cm^−1^. The intensity of the peak increased by the increase in carvacrol content from 5 to 10% in carvacrol-loaded PLA fibers. Similarly, the intensity of peaks at 1453, 1382, 870, and 756 cm^−1^ increased in carvacrol and geraniol-loaded PLA fibers. Bands of carvacrol molecules at 1458, 1382, 870, and 756 cm^−1^ were observed in the spectrum of carvacrol-loaded PLA fibers at 1456, 1383, 871, and 755 cm^−1^, respectively. Bands of geraniol molecules at 1440, 1376, 831, and 777 cm^−1^ were observed in the spectrum of geraniol-loaded PLA fibers at 1455, 1383, 871, and 755 cm^−1^, respectively. The peak shifts suggest the incorporation of carvacrol and geraniol in the electrospun PLA fibers. In addition, the intensity of these peaks increased with the carvacrol and geraniol content, which shows the presence of carvacrol and geraniol in PLA fibers. The overall spectra confirmed the presence of carvacrol and geraniol in monoterpenoid-loaded PLA fibers, which are in agreement with previous studies [51,52].

### 3.4. Antibacterial Assessment

The antibacterial activity of carvacrol and geraniol against *E. coli* and *S. epidermidis* strains have been demonstrated. Monoterpenoid-loaded electrospun webs exhibited potential concentration-related antibacterial effects against both Gram-positive and Gram-negative bacteria (Table 3). Generally, both strains responded to the tested monoterpenoids. As shown in Table 3, carvacrol-loaded fibers with the largest inhibition zone (5.26 ± 0.05 and 4.20 ± 0.17 cm) exhibited a significant antibacterial property against the tested bacteria compared to geraniol-loaded fibers. The Control sample and G5 exhibited no antibacterial actively, as no inhibition zone was observed on the two tested bacteria. In agreement with previous reports [28], although nanofibers containing more monoterpenoids had a higher antibacterial effect, carvacrol showed a stronger effect in both 5 and 10 wt% compared to that of geraniol. On the other hand, both monoterpenoids showed a stronger antibacterial activity against the *S. epidermidis* strain. A possible reason could be the presence of an additional layer of lipids in the cell wall of the Gram-negative strain, which acts as a permeability barrier. Overall, the inhibition zone diameter tends to increase in proportion to the increasing concentration of the monoterpenoids. As previous studies have shown, carvacrol and geraniol appear to have distinct modes of action against various strains and display potent antibacterial activity due to the chemical structure of monoterpenoids [53]. In particular, monoterpenoids that contain a high percentage of phenolic compounds such as carvacrol generally show the strongest antibacterial properties against food-borne pathogens [54]. However, not all components are as active, as carvacrol and the presence of an hydroxyl group in the phenolic ring are crucial [29]. These phenolic compounds help enable an antimicrobial effect similar to that of carvacrol by rupturing of the cytoplasmic membrane caused by active transport and electron flow, causing coagulation of the cell contents and cell death [29,55]. Previously, geraniol-loaded polymeric nanoparticles were found to inhibit *E. coli* growth at 0.2 wt% [56], and carvacrol-loaded chitosan nanoparticles were used to maintain the microbial quality of fresh-cut vegetables.

**Figure 5:**
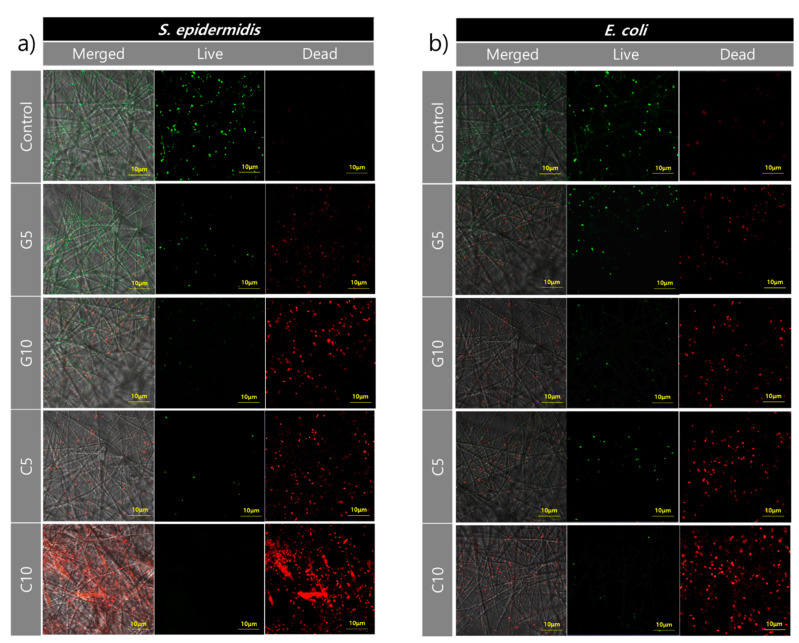
Fluorescence microscopic images of a Live/Dead bacterial cell viability assay. Representative images of (**a**) *S. epidermidis* and (**b**) *E. coli* cultured on carvacrol or geraniol-loaded PLA nanofiber webs after 24 h of incubation (green refers to live bacteria and red refers to dead bacteria).

To confirm the potential antibacterial activities of carvacrol- and geraniol-loaded electrospun webs against bacteria strains, fluorescent microscopy images were used. SYTO 9 and PI were applied for the staining of live and dead bacteria. SYTO 9 stained the live bacteria and portrayed a green fluorescence, while the dead bacteria with damaged membranes were stained by PI and displayed a red fluorescence. As the confocal microscopy images (Figure 5) demonstrate, many live *E. coli* and *S. epidermidis* bacteria (green) were attached on the Control surfaces, while almost no dead bacteria (red) were observed. In contrast, a large number of dead bacteria (red) were observed on monoterpenoid-loaded webs. As shown in Figure 5. the intensity of the red fluorescence color was highest for C10 compared to the rest, indicating a higher antibacterial effect when the concentration of the monoterpenoid was increased. For both *E. coli* and *S. epidermidis* strains, the overall intensity of red color was as follows: C10 > C5 > G10 > G5. Both carvacrol and geraniol showed a stronger antibacterial activity against the *S. epidermidis* strain. This was in agreement with the results of the inhibition zone assay in which the largest inhibition zone was observed in the C10 sample for *E. coli* and *S. epidermidis* bacteria. The results imply that monoterpenoid-treated electrospun webs show powerful antibacterial properties against Gram-positive *S. epidermidis* bacteria compared to Gram-negative *E. coli*.

**Figure 6:**
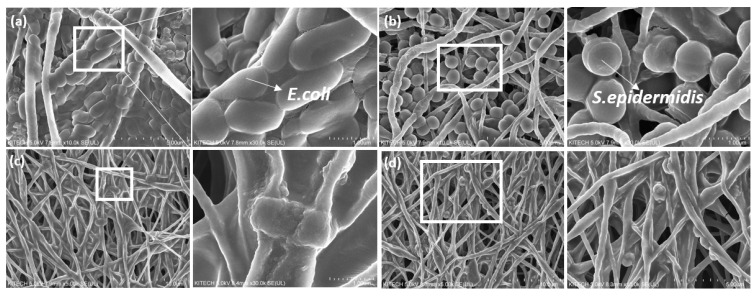
SEM images of bacterial adhesion phenomenon depending on antimicrobial activities: (**a**) neat PLA control against *E. coli*; (**b**) neat PLA control against *S. epidermidis*; (**c**) C10 against *E. coli*; and (**d**) C10 against *S. epidermidis* after 24 h of incubation.

SEM images showed differences in bacteria cell features and content between the PLA control sample and 10 wt% carvacrol-loaded electrospun web (C10). In the control sample, high bacterial adhesion was observed, and bacterial cells remained undamaged (rod- and coccus-shaped) with a smooth surface, as shown in Figure 6a,b. However, structural changes were observed in the C10 bacteria cell samples (Figure 6c,d), and the decrease in the number of adhered bacteria to the surface of C10 was also confirmed via SEM images. The major changes in cell structure and decrease in adhesion may be the result of carvacrol intrusion between the membrane lipid bilayers of bacteria cells, damaging membrane fluidity.

From previous antibacterial tests such as inhibition zone tests and live/dead bacterial cell assay, C10 was the most efficient antibacterial agent in this study. However, it was difficult to evaluate precise antibacterial effects as G5, G10, and C5 exhibited similar or little antibacterial activity. In order to analyze quantitatively the antibacterial activities of all PLA control, C5, C10, G5, and G10 samples against *E. coli* and *S.* *epidermidis*, growth curves were analyzed using OD_600_ depending on the 72 h incubation time. The inhibition % was calculated by using Formula (1)

(1) $$Inhibition\;\left(\%\right)=\left(1-\frac{OD600\;of\;the\;treated\;sample\;at\;incubation\;time}{OD600\;of\;the\;PLA\;control\;at\;incubation\;time}\;\right)\times \;100$$

**Figure 7:**
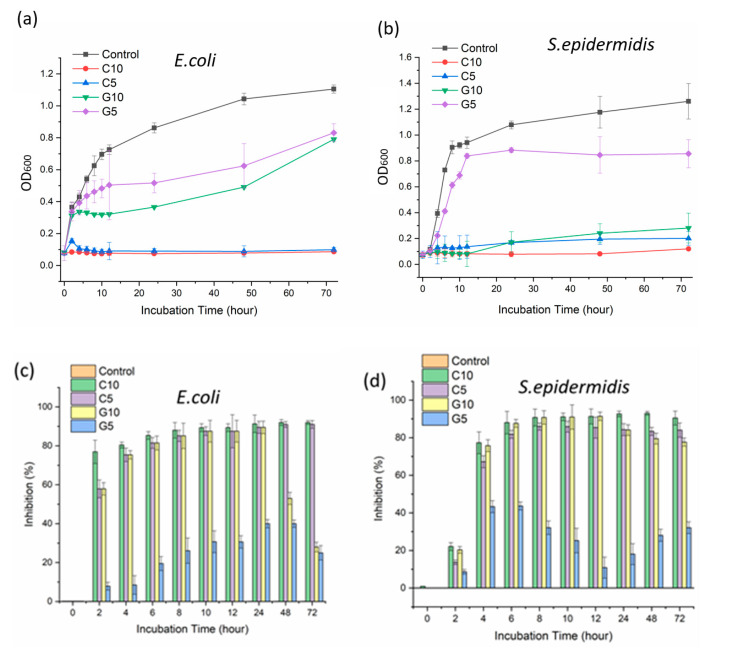
Bacterial growth kinetic curves of carvacrol or geraniol-loaded PLA nanofibers for a 72 h incubation time against (**a**) *E. coli* and (**b**) *S. epidermidis*, and (**c**) inhibition percentages (%) against *E. coli* and (**d**) *S. epidermidis*.

The growth of *E. coli* and *S. epidermidis* was more significantly inhibited by carvacrol-loaded PLA nanofiber webs, as shown in Figure 7, compared to the PLA control. Obvious dose-dependence antimicrobial performances of carvacrol or geraniol-loaded PLA nanofiber webs were observed. Furthermore, for C5, C10, and G10, the growth of *S.* *epidermidis* was almost thoroughly restrained within 72 h over 80% inhibition. C10 exhibited good antibacterial performance against both *E. coli* and *S.* *epidermidis* with over 92% of inhibition within 24 h (Figure 7c,d). The antibacterial performance against both *E. coli* and *S. epidermidis* was as follows: C10 > C5 > G10 > G5. The growth of *E. coli* was almost completely inhibited with the utilization of C10 within 2 h, whereas *S. epidermidis* was required until 4 h. For C5, G5, and G10 samples against *S.* *epidermidis*, the values of OD_600_ were increased after 24 h of incubation, whereas the values of OD_600_ against *E. coli* were increased after 48 h of incubation. This is mainly attributed to the different peptidoglycan layer structures in the cell wall of *E. coli* and *S. epidermidis*. *S. epidermidis* has a thicker peptidoglycan layer with a higher cross-linking degree in the cell wall compared with that of *E. coli* [57]. Therefore, the total inhibition of *S.* *epidermidis* required a higher concentration of monoterpenoids than *E. coli* did. Carvacrol and geraniol showed potent antibacterial activity against *E. coli* and *S. epidermidis*.

## 4. Conclusions

This study used the emulsion electrospinning method to fabricate electrospun webs loaded with monoterpenoid for antibacterial effects. Both webs containing one type of monoterpenoid, carvacrol or geraniol, demonstrated antibacterial properties against *E. coli* and *S. epidermidis* strains, although the carvacrol-loaded nanofibers exhibited a higher antibacterial effect compared to geraniol due to the difference in chemical structure of the components. The results showed that increasing the amount of monoterpenoids loaded into the electrospun fiber also increased the antibacterial activity of the fibers. Furthermore, both carvacrol and geraniol monoterpenoids exhibited a higher antibacterial effect on Gram-positive bacteria compared to Gram-negative bacteria. Thus, this research successfully demonstrated that monoterpenoids, when loaded in electrospun PLA nanofibers, have significant potential to be influential antibacterial agents.

## Figures and Tables

**Table 1:** Substrates’ sample description and characteristics.

Code	Description
C	Carvacrol
G	Geraniol
control	Electrospun neat PLA nanofibers
C5	Electrospun PLA nanofiber with addition of 5 wt% carvacrol of PLA solution
C10	Electrospun PLA nanofiber with addition of 10 wt% carvacrol of PLA solution
G5	Electrospun PLA nanofiber with addition of 5 wt% geraniol of PLA solution
G10	Electrospun PLA nanofiber with addition of 10 wt% geraniol of PLA solution

**Table 2:** Analysis (wt%) of electrospun PLA webs and monoterpenoids on thermogravimetric analysis (TGA).

		Weight Loss	Sample						
			C	G	Control	C5	C10	G5	G10
Distribution of polymer degradation and monoterpenoids evaporation	Step1	Temp. range (°C)	27–133	22–133	257–375	24–118	27–119	27–103	27–112
		%	99.26	99.77	96.26	11.10	19.98	7.14	16.48
	Step2	Temp. range (°C)	-	-	-	163–378	257–371	143–369	145–342
		%	-	-	-	89.4	77.67	80.55	89.40

**Table 3:** Inhibition zones of the electrospun webs against two bacteria strains: *E. coli* and *S. epidermidis*.

	Sample				
	Control	C5	C10	G5	G10
*E. coli*	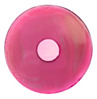	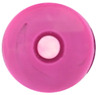	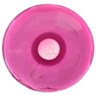	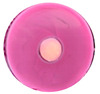	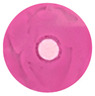
Inhibition zone diameters (mm)	-	30.6 (± 0.4)	36.7 (± 0.8)	-	26.6 (±0.8)
*S. epidermidis*	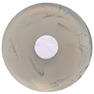	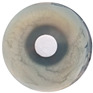	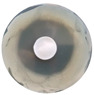	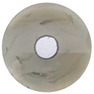	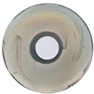
Inhibition zone diameters (mm)	-	42.0 (±1.7)	52.6 (±0.5)	-	31.3 (±0.5)
